# Outcomes within a year following first ever stroke in Tanzania

**DOI:** 10.1371/journal.pone.0246492

**Published:** 2021-02-11

**Authors:** Kezia Kodawa Tessua, Patricia Munseri, Sarah Shali Matuja

**Affiliations:** 1 Department of Internal Medicine, Muhimbili University of Health and Allied Sciences, Dar es Salaam, Tanzania; 2 Department of Internal Medicine, Catholic University of Health and Allied Sciences, Mwanza, Tanzania; Hospital Dr. Rafael A. Calderón Guardia, CCSS, COSTA RICA

## Abstract

**Background:**

Stroke contributes to a significant proportion of deaths and disability worldwide, with a high fatality rate within 30 days following a first ever stroke. We describe the outcomes within one year among patients who succumbed a first ever stroke and survived the first 30 days.

**Methods:**

Participants were patients who survived after 30 days from succumbing a first ever stroke admitted at the Muhimbili University of Health and Allied Sciences Academic Medical Center. Stroke survivors or their next of kin were contacted at one year after succumbing a first stroke to determine the outcomes. We assessed participants’ vital status and level of disability using the modified Rankin scale. Assessment on utilization of stroke secondary preventive measures among survivors was done by an interviewer-based questionnaire that assessed the number of times participants attended follow up clinics, medication refill and adherence. Participants were examined for waist-hip ratio, body mass index and blood pressure. Cholesterol levels were assessed at one year post first stroke for survivors. Outcomes were summarized as proportions, survival at one year was estimated by using the Kaplan Meier analysis and Cox regression analysis was performed to determine for predictors of mortality.

**Results:**

We recruited 130 first stroke survivors. Mortality within one year was 53/130 (40.8%) and disability rate measured by Modified Rankin Scale with scores of 3–5 was 29/77 (37.7%) among survivors. Factors associated with mortality were residual disability HR = 8.60, {95% CI (1.16–63.96)}, severe stroke, HR = 2.67 {95% CI (1.44–4.95)} and residing in Dar-es-Salaam HR = 2.15 {95% (CI 1.06–4.36)}. Non-adherence rates to antihypertensives, antiplatelets and statins was 11/73 (15.1%), 9/23 (39.1%) and 18/22 (81.8%) respectively. Attendance rates of follow-up clinics among all survivors and physiotherapy among survivors with disability are 45/77 (58.4%) and 16/29 (55.2%) respectively.

**Conclusions:**

The mortality and disability rates within a year following a first ever stroke among 30 days stroke survivors is high. Secondary stroke preventive measures should be enhanced to mitigate stroke adverse outcomes. Community outreach programs could be useful interventions in preventing the adverse outcomes of stroke.

## Background

Globally there were 13.7 million new stroke cases for the year 2016 [1]. While the incidence of stroke has been decreasing by 42% over the past four decades in high income countries, low and middle income countries have experienced a 100% increase and contribute to over 80% of the stroke burden globally [2]. In Africa, the incidence of stroke ranges between 25 to 250 per 100,000 population [3]. In a three-year Tanzanian population based study conducted between 2004 and 2006, the crude stroke incidence was 107.9 and 94.5 per 100,000 for urban and rural areas respectively [4]. Hospital admissions from stroke have increased tremendously from 2.9 per 100,000 in 1974 to 202.2 per 100,000 in 2017 for Tanzania [5].

Modifiable risk factors such as hypertension, diabetes mellitus, obesity, dyslipidemia, cigarette smoking, alcohol consumption, psychosocial stress and rheumatic mitral stenosis [2] have contributed to more than 90% of stroke causes [5].

The outcomes of stroke are either death or survival [6, 7]. Survival with disability can either progress to death or result in poor quality of life [8, 9].

Stroke accounted for 5.5 million deaths and was the second leading cause of death globally in 2016 [1]. Stroke contributed to an overall mortality of 12% in Ethiopia between 2012 and 2014 [10] and 50% at 90 days following first ever stroke in Tanzania in 2017 [11].

Globally stroke led to 116.4 million disability adjusted life years (DALYs) in the year 2016 [1]. The risk for disability after a first stroke in Europe is estimated to be 20% [12] whereas for Tanzania, post stroke severe disability was 56% and 49% for ischemic and hemorrhagic stroke respectively [11]. Prompt and effective management of hypertension, diabetes and thrombosis reduce the risk of stroke recurrence while rehabilitation reduces disability [13]. Behavioral changes such as smoking cessation and reduction in alcohol consumption are additional strategies for stroke prevention [14]. We describe outcomes within one year of patients surviving 30 days following first ever stroke in Tanzania.

## Methods

This cohort study enrolled patients who survived after 30 days from succumbing a first ever stroke admitted at Muhimbili University of Health and Allied Sciences Academic Medical Center (MAMC) between June 2018 to January 2019 at a year post stroke. MAMC is a specialized hospital that receives walk in and referral patients from all over the country. The hospital offers medical care in several disciplines including neurology and neurosurgery.

### Data collection

The investigator (KM) and co-investigator (SM) identified 136 patients out of the 369 patients who survived after 30 days post first ever stroke who were admitted at MAMC between June 2018 and January 2019. There were 233 patients who died within 30 days from first ever stroke [15] leaving 136 stroke survivors eligible for one year follow up. We were unable to contact six participants or their next of kin and contacted and recruited 130 participants/next of kin. Out of the 130 participants who were enrolled, 58 participants managed to individually respond to phone calls while the remaining 72 participants their next of kin responded to phone calls on their behalf. Stroke survivors or their next of kin were contacted by phone to inquire on the vital status one year following first ever stroke. Information on vital status was recorded in a study specific structured questionnaire. Survivors were requested to take part in a follow up study and all who agreed were requested to attend for a clinic visit at MAMC. Written informed consent was obtained prior to enrollment of participants in to the follow up study. There were 40 participants who resided out of Dar-es-Salaam and 34 participants from Dar-es-Salaam who were out of the city during the study period being cared for by their relatives who lived up country. These 74 participants were followed up by trained health care personnel at their nearest health centers who assisted in collecting data. Information collected from stroke survivors included demographic characteristics including age, sex and employment status. History of hypertension, diabetes mellitus, HIV infection, cigarette smoking and alcohol consumption before and after the first stroke were obtained. Informal employment was defined by international labor organization (ILO) as economic activities that are not or are insufficiently covered by formal law or practice arrangements [16]. Waist circumference measurements were recorded in centimeters in a standing or supine position at the midpoint between the lowest palpable rib and anterior superior iliac spine. A waist circumference of ˂ 94cm and ˂ 80cm for males and females respectively was considered normal. Hip circumference measurement was taken around the widest point of the hips. The waist hip ratio was calculated by dividing the waist circumference in centimeters (cm) to the hip circumference in centimeters (cm). Abdominal obesity was defined as waist hip ratio ≥ 0.9 for males and ≥ 0.85 for females [17]. Weight was measured using a SECCA weighing scale to the nearest 0.5Kg. Height was measured using a height measuring rod and recorded to the nearest 0.5cm. Body mass index (BMI) was calculated as weight in kilograms (kg) divided by height in meters (m) squared. BMI was classified as underweight ˂18.5Kg/m^2^, normal 18.5–24.9Kg/m ^2^, overweight 25–29.9Kg/m^2^ or obese ≥ 30Kg/m^2^. Blood pressure was measured in sitting position using an automated sphygmomanometer MICROLIFE™. Hypertension was defined as systolic blood pressure ≥ 140mmHg and/ or diastolic blood pressure of ≥ 90mmHg [18]. Each participant was requested to provide 5mls sample of whole blood for Cholesterol levels that was analyzed by a chemistry analyzer model Cobas Integra 400 Plus. Hypercholesterolemia was defined as total cholesterol levels > 5.2mmol/L. Fingerpick capillary sample was obtained for HIV testing using SD Bioline (Standard Diagnostic Inc, Korea), reactive tests were confirmed using Uni-Gold assay (Trinity, UK). Blood glucose was assessed from finger prick sample using a standard GLUCOPLUS™ glucose meter. Blood glucose levels of ≥7 mmol/L and ≥ 11.1mmol/L for fasting and random values respectively were considered diabetes. All the instruments used for measurements were calibrated. Blood pressure, waist and hip circumference, height and weight measurements were in accordance to guidelines [17, 18] that were applied consistently and counterchecked before entering data into dataset for each participant. Competent trained laboratory staff processed and analyzed the study samples at the Jakaya Kikwete Cardiac Institute laboratory that has a quality control team and standard operating procedures for testing and analyzing samples. All the laboratory test results were verified by a senior laboratory scientist.

Each participant’s baseline information including: the first ever stroke subtype, severity, disability status, clinical and laboratory findings during the first stroke were obtained from a study specific data set [15].

First stroke subtype was confirmed using brain imaging by either a non-contrast Computerized Tomography (CT) scan model GE Healthcare Optima CT660 SE, or Magnetic Resonance Imaging (MRI) model GE SIGNA CREATOR 1.5 TESLA [15]. Stroke severity was assessed during the time of first ever stroke using the National Institutes of Health Stroke Scale (NIHSS) [15] and grouped as minor stroke with NIHSS scores of 1–4, moderate stroke with NIHSS scores of 5–20 and severe strokes with NIHSS scores of 21–42 [19]. The levels of baseline stroke severity were used to assess predictors for outcomes within one year.

Disability at one year was assessed using the Modified Rankin Scale (mRS) [20]. We have classified survival with mild disability as functionally independent state with mRS˂3. Survival with moderate to severe disability and functionally dependent state defined by mRS 3–5 (moderate disability as mRS = 3 while severe disability as mRS = 4–5). Baseline disability status in this study was defined as disability status at 30 days after first stroke.

### Ethical approval and consent to participate

Ethical clearance was obtained from Muhimbili University of Health and Allied Science institutional review board approval reference number DA.287/298/01A/. Written informed consent was obtained from all study participants or their next of kin prior to enrollment, including consent to HIV testing. Participants who were found to have uncontrolled cardiovascular risk factors were counselled and treated according to standard treatment guidelines.

### Data analysis

Data analysis was performed using SPSS version 23.0. The outcomes of stroke were summarized as proportions. Survival at one year for stroke participants was estimated using Kaplan Meir analysis. Comparison in differences in survival at one year by age groups, stroke subtype and severity was computed by Log rank test. Cox regression method was performed to assess for predictors of mortality at one year, variables with a p value <0.2 in univariate analysis were entered in to the multivariate analysis model and a p value <0.05 was considered as statistically significant.

## Results

**Fig 1:**
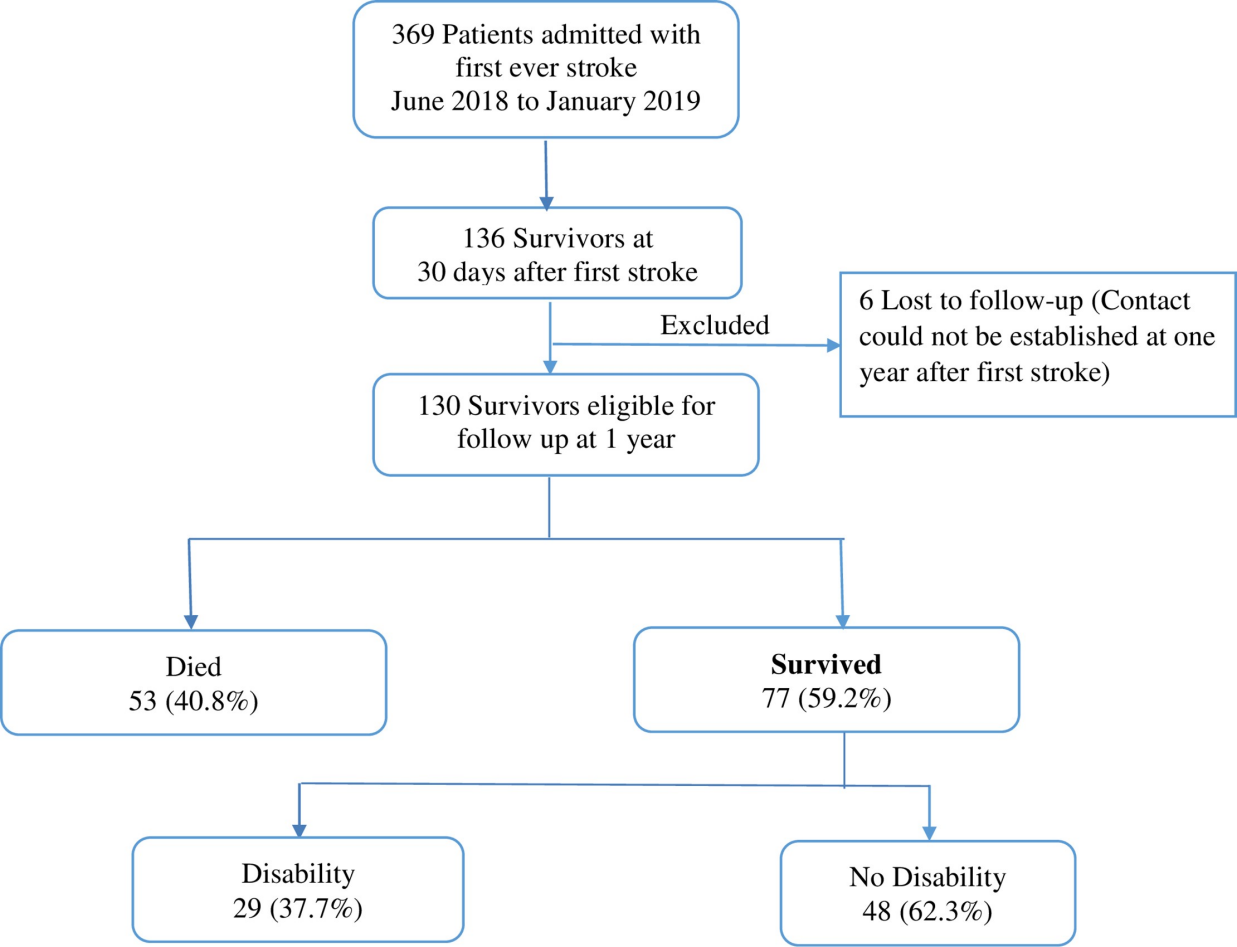
Consort diagram.

A total of 136 patients survived for 30 days following a first ever stroke. At one year of follow up, we were able to contact 130 (95.6%) survivors or their next of kin, six (4.4%) patients and their next of kin were not reachable by phone and were excluded. Of the 130 evaluable participants, males and females were 47(36.2%) and 83(63.8%) respectively. There were 53/130 (40.8%) who died within one year of succumbing a stroke, as shown in Fig 1.

The mean age and standard deviation of the 130 first ever stroke survivors was 54 ± 16 years. The mean age for the 53 participants who died within one year was 57 ± 17 years while mean age for the 77 participants who survived up to one year was 51 ± 14 years. A significant proportion of participants who died within a year of succumbing a stroke were aged > 45 years 37/74 (50%) vs 16/56 (28.6%) p = 0.014 and had informal employment 44/92(41.1%) vs 1/15(6.7%), p = 0.01 as summarized in Table 1.

**Table 1:** Baseline demographic characteristics of first stroke survivors.

Demographics	Died	Survived	P value
	n = 53	n = 77	
Age group			
≤45 years	16 (28.6%)	40 (71.4%)	0.014
>45 years	37 (50%)	37 (50%)	
Male	22 (46.8%)	25 (53.2%)	0.292
Employment			
Formal	1 (6.7%)	14 (93.3%)	0.020
Informal	44 (41.1%)	63 (58.9%)	
Insured	18 (40.9%)	26 (59.1%)	0.999
Residence			
Dar	42(46.7%)	48(53.3%)	0.04
Upcountry	11(27.5%)	29(72.5%)	

Majority of participants had moderate to severe disability at baseline, 112/130 (86.2%). Among 53 participants who died, 48 participants could perform brain CT scan during admission for the first ever stroke and there were 34(70.8%) who had ischemic stroke while 9(18.8%) had hemorrhagic stroke. A higher proportion of participants with severe stroke at baseline died within one year as compared to those who survived (21/53, 39.6% and 9/77, 11.7%), p value < 0.001. Other clinical characteristics are presented in Table 2.

**Table 2:** Baseline clinical characteristics and stroke related factors of first stroke survivors.

Clinical history	Died	Survived	Loss to follow up	P value
	n = 53	n = 77	n = 6	
Hypertension	48 (90.6%)	75 (97.4%)	5 (83.3%)	0.137
Diabetes mellitus	12 (22.6%)	11 (14.3%)	1 (16.7%)	0.469
HIV	3 (5.7%)	6 (7.8%)	0 (0.0%)	0.713
First stroke subtype *				
Ischemic	34 (70.8%)	32 (43.8)	3 (50%)	0.014
Hemorrhagic	9 (18.8%)	30 (41.1%)	1 (16.7%)	0.025
Mixed	2 (4.2%)	0 (0.0%)	0 (0.0%)	0.188
Normal	3 (6.3%)	11 (15.1%)	2 (33.3%)	0.105
First stroke severity NIHSS				
Minor	2 (3.8%)	9 (11.7%)	0 (0.0%)	0.202
Moderate	30 (56.6%)	59 (76.6%)	6 (100%)	0.013
Severe	21(39.6%)	9 (11.7%)	0 (0.0%)	< 0.001
30 Day disability status mRS				
Mild disability(mRS<3)	1(1.9%)	17 (22.1%)	0 (0.0%)	
Moderate to severe disability(mRS = 3–5)	52 (98.1%)	60 (77.9%)	6 (100%)	0.002

*Missing data on first stroke subtype for 9 (6.6%) of participants (5 died and 4 alive) as they did not perform brain CT scan or MRI due to financial constraints.

NIHSS- National Institutes of Health Stroke Scale, mRS- Modified Rankin Scale, HIV-Human Immunodeficiency Virus

**Fig 2:**
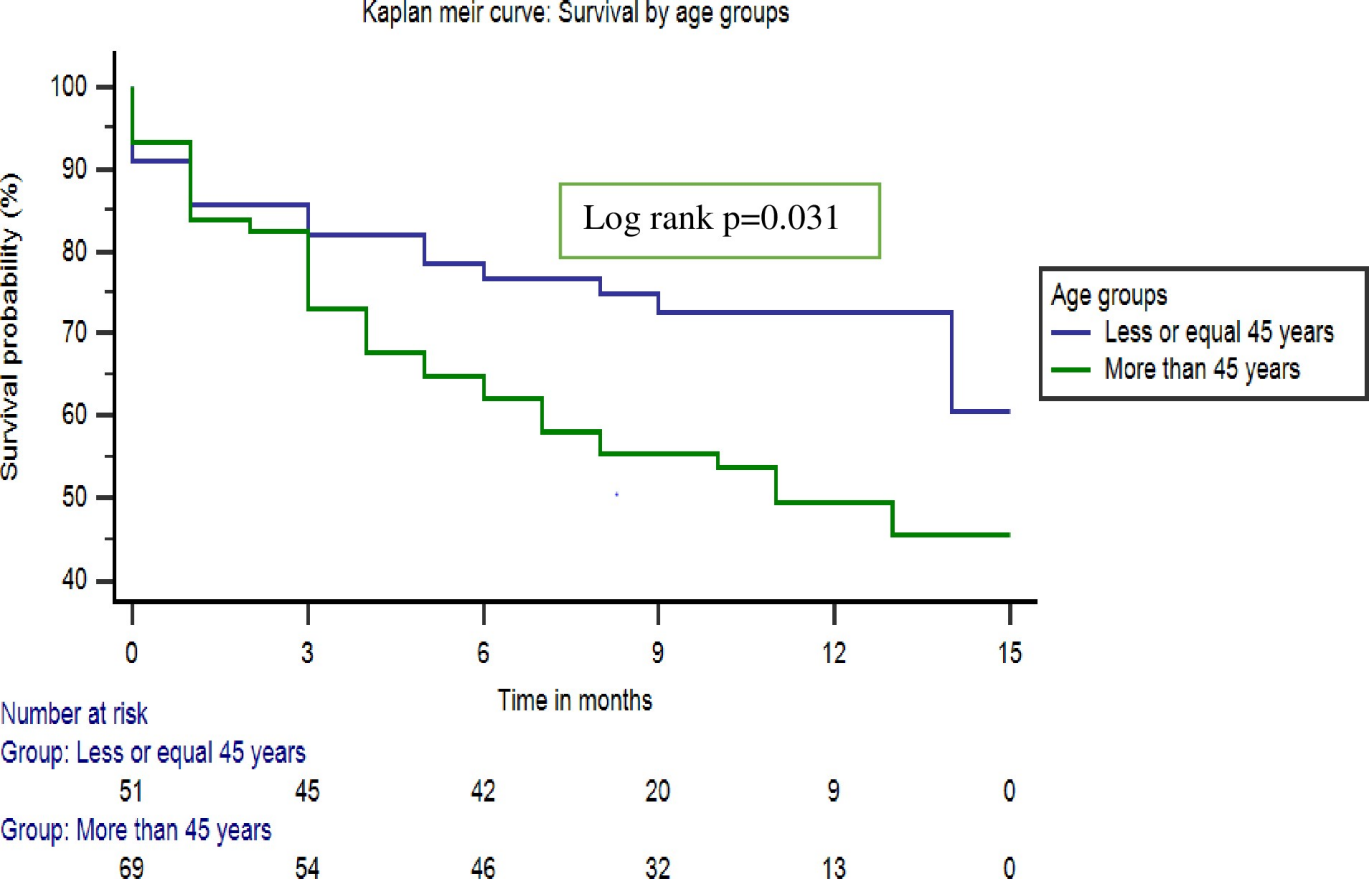
Kaplan Meier curve: One-year survival by age groups.

Participants who were 45 years or below were more likely to survive at 1 year following a stroke, {HR: 1.8, (95% CI 1.06–3.22)} compared to participants above 45 years, {HR: 0.54, (95% CI 0.31–0.95)}, p = 0.031, as illustrated in Fig 2.

Predictors of mortality included: severe stroke, {HR: 2.67, (95% CI; 1.44–4.95)}, presence of disability at 30 days after first stroke, {HR: 8.60, (95% CI: 1.16–63.96)} and residing in Dar-es-Salaam region, {HR: 2.15, (95% CI: 1.06–4.36)} (Table 3).

**Table 3:** Cox proportional hazard for predictors of mortality at one year after first ever stroke.

Variable	n/N(%)	HR(95% CI)	p-value	Adjusted HR(95% CI)	p- value
Age group					
≤45 years	16/56(28.6%)	1			
˃45 years	37/74(50%)	1.858(1.033–3.342)	0.039	1.553(0.825–2.926)	0.173
Sex					
Male	22/47(46.8%)	1			
Female	31/83(37.3%)	0.816(0.472–1.409)	0.468		
Stroke subtype					
Hemorrhagic stroke	9/39(23.1%)	1			
Ischemic stroke	34/66(51.5%)	2.3(1.236–4.304)	0.009	1.75(0.669–4.448)	0.256
First stroke severity					
Minor	2/11(18.2%)	1			
Moderate	30/89(37.7%)	2.1(0.501–8.793)	0.31		
Severe	21/30(70%)	6.561(1.53–28.13)	0.011	2.669(1.440–4.946)	0.002
Residency					
Upcountry	11/40(27.5%)	1			
Dar	42/90(46.7%)	2.06(1.056–4.016)	0.034	2.148(1.058–4.358)	0.034
30-Day disability					
Mild disability	1/18(5.6%)	1			
Moderate-severe disability	52/112(46.4%)	10.47(1.45–75.88)	0.02	8.601(1.159–63.958)	0.035
Hypertension					
Yes	48/123(39%)	1			
No	5/7(71.4%)	2.373(0.943–5.973)	0.067	2.338(0.813–6.723)	0.115
Diabetes					
No	41/107(38.3%)	1			
Yes	12/23(52.2%)	1.4(0.746–2.705)	0.285		
HIV infection					
No	50/121(41.3%)	1			
Yes	3/9(33.3%)	0.716(0.223–2.296)	0.574		

At one year, 29/77(37.7%) participants who survived had moderate to severe disability with mRS scores of 3–5 while 48/77(62.3%) survived with mild disability with mRS score of less than 3. Among participants with moderate to severe disability, 14/29 (48.3%) had moderate disability (mRS = 3) and 15/29 (52.7%) had severe disability (mRS = 4–5). There were 28/29 (97%) of participants with moderate to severe disability who were unable to resume to work.

Among one year first stroke survivors, non-adherence rates to antihypertensive, antiplatelet and statins medications were 11/73 (15.1%), 9/23 (39.1%) and 18/22 (81.8%) respectively. Among 29 patients who recovered with moderate and severe disability at one year, 13 (44.8%) had not undergone any physiotherapy (Table 4).

**Table 4:** Secondary preventive measures uptake among stroke survivors at one year.

Variable	N	Percentage
Antihypertensive medication adherence* (N = 73)		
Yes	62	84.9%
No	11	15.1%
Antiplatelet use (N = 23)		
Yes	14	60.9%
No	9	39.1%
Statins use (N = 22)		
Yes	4	18.2%
No	18	81.8%
Attending clinic (N = 77)		
Yes	45	58.4%
No	32	41.6%
Physiotherapy (N = 29)		
Yes	16	55.2%
No	13	44.8%
Knowledge on stroke recurrence (N = 77)		
Yes	45	58.4%
No	32	41.6%

N = number of participants utilizing secondary preventive measures

*Missing data: 2 participants were not prescribed antihypertensive medication

Of the 77 stroke survivors at one year, 32 (41.6%) lacked knowledge that stroke could recur and 75 (97.4%) were hypertensive. Blood pressure measurements were obtained in 73/75(97.3%) participants, two participants could not get to MAMC and nearby health centers did not have functioning blood pressure machines. Among the 73 participants, 47(64.4%) had uncontrolled hypertension, with mean and standard deviation of 159.1 ± 19.6mmHg for systolic blood pressure and 102 ± 12.2mmHg for diastolic blood pressure.

A total of 56/70 (80%) had higher than normal waist hip ratio with a mean waist hip ratio and standard deviation of 0.97 ± 0.14. Among the 70 participants who had waist hip ratio measured, 22 were male and 48 were female, 17/22 and 39/48 had abdominal obesity with waist hip ratios and standard deviations of 0.94 ± 0.09 and 0.94 ± 0.15 for males and females respectively.

BMI was computed for 44 participants others could not have this measurement as they were disabled or were from upcountry and could not get to nearby centers where weight and height could be measured. Out of the 44 participants 18(40.9%) were obese and 17(38.6%) were overweight.

Total cholesterol levels were above normal in 22/56 (39.3%) Table 5. Cholesterol levels could not be checked for all as some participants were at areas where transporting samples to Dar-es-Salaam was limited due to long distance.

**Table 5:** Clinical examination and laboratory characteristics among stroke survivors at one year.

Variable	N	Percentage
BP control* (N = 73)		
Yes	26	35.6%
No	47	64.4%
Waist hip ratio control* (N = 70)		
Yes	14	20%
No	56	80%
BMI* (N = 44)		
Normal	9	20.5%
Overweight	17	38.6%
Obese	18	40.9%
Total cholesterol levels(N = 56)		
Normal	34	60.7%
High	22	39.3%

N- Number of participants whose measurements were taken at one year, BP-Blood pressure, BMI-Body mass index

*Missing: could not get to centers where BP measurements, waist hip measurements could be taken

*Missing BMI: Could not get to centers with weighing or height scale or were disabled

## Discussion

We followed up first ever stroke patients who survived the first thirty days of succumbing a stroke and observed a high mortality rate of 40.8% within a year. Mortality was linked with a severe stroke, presence of moderate to severe disability at 30 days, and residing in Dar-es-Salaam.

In this study we observed that, patients with baseline moderate to severe disability had a nine fold increased risk of dying within a year of first ever stroke compared to patients with mild disability. Patients with severe stroke experience worse disability that results in loss of function with risk of complications such as aspiration, deep vein thrombosis, pressure sores and depression that increase the mortality risk [21, 22].

Patients with disability may have further neurological worsening, comorbidities or complications that may not be easily noticed by their caregivers that could contribute to death as a result of delayed health care seeking.

Severe disability is linked to depression and reduced motivation that may result into reduced adherence of post stroke management plans eventually predisposing patients with disability to death [23]. Stroke severity and disability have been shown to be important factors contributing to the one year mortality of stroke patients in high income countries [24].

We observed a relatively lower prevalence of disability from stroke compared to what was observed more than a decade ago in northern Tanzania, (37.7% vs 46.5%) [25]. This could be due to increased socio-economic status and access to physiotherapy over time. However, the disability rate in this study was higher compared to high income countries (37.7% vs 28.8%) [26].

In this study 97% of the patients with disability were unable to return to work out of which a third would be considered to be in the prime time of building the economy as they were aged 45 years or less.

In this study patients with disability were unable to attend for physiotherapy sessions due to lack of finances to pay for transportation and consultation for the services and lack of care takers to assist the patient. Stroke and its associated disability management depend on a multidisciplinary team approach. Outreach services and home-based care could be important approaches in reducing patient costs, improving function for patients with disability, assessment and management of comorbidities and overall reduction in mortality.

Mortality within a year of stroke was twice as high for participants residing in Dar-es-Salaam, an urban city in Tanzania compared to residents from upcountry. This is surprising based on the fact that Dar-es-Salaam has a relatively higher number of health care facilities that are better equipped with staff, equipment and services compared to the rest of the country. The low socio-economic status among the study participants could have contributed to delayed access to health care and observed mortality based on the fact that; 83% of our participants were informally employed, were more likely to be the income generators for the family and therefore lacked financial sustainability, and two thirds did not have health insurance. Out of pocket payment for health services hinders timely health seeking behavior for stroke survivors [27]. Interestingly higher mortality rates have also been observed in the urban compared to rural areas of Asia (42% vs 30%) due to free annual lipid level screening offered for rural residents hence timely intervention for abnormal lipid levels and higher lipid levels were observed in urban area residents than rural residents [28, 29].

Age above 45 years and severe stroke inversely correlated with one year survival following a first ever stroke as was observed in both low, middle and high countries [30, 31]. Patients who sustained hemorrhagic stroke had better one-year survival compared to patients who sustained ischemic stroke. In this study almost half (53.8%) of the patients who were young, aged 45 years or less sustained hemorrhagic stroke that could have contributed to survival. Ischemic stroke patients are known to have better one year survival compared to patients with intracerebral hemorrhage due to availability of interventions for acute ischemic stroke in high income countries that results in reduced disability and complications [32].

More than a half of our study participants had uncontrolled hypertension, were overweight or obese or had abnormal waist hip ratio measurements. This could be due to lack of adherence for medications for hypertension, lipid abnormalities and antiplatelets. More than a third of participants were not attending follow up clinics regularly and this led to high levels of uncontrolled blood pressure levels due to lack of proper monitoring and dose adjustments for antihypertensive medications. In low and middle income countries similar challenges are experienced in controlling stroke risk factors where in South Africa only 8% of stroke survivors were adhering to antihypertensive medications and only about half were taking antiplatelets at 3 months follow up after stroke. Reasons for non-adherence in these studies are lack of knowledge about hypertension being a chronic condition, costs for medications and lack of physical assistance to reach health care facilities and alcohol abuse [33, 34].

More than a third of the study participants or their care takers were unaware that stroke could recur, lack of knowledge could contribute towards delay in timely health seeking behavior and adherence to medications. The barriers to utilization of secondary stroke prophylaxis and adherence to statins, antiplatelets, antihypertensives and attendance to follow up clinics and physiotherapy services among the participants were financial constraints and long distance to health care services. There should be proper counselling to stroke patients and their caretakers on adverse outcomes of stroke like recurrence and death. Proper adherence to the measures for control of cardiovascular risk factors to eliminate the outcomes should be emphasized by health care workers through health education.

The cost and long distance incurred by the patients can be mitigated if tertiary hospitals provide outreach services and build capacity of primary health centers to provide rehabilitation services and long term management of stroke patients. Caretakers could also be trained on how to provide physiotherapy especially for patients who have disability and are unable to obtain transportation to a health care facility.

The estimate of disability could be underestimated due to the fact that we could not obtain data from participants who died as a result of severe disability. Likewise, we were unable to obtain information on utilization of secondary preventive measures for patients who died therefore limiting the ability to ascertain if these factors were associated with mortality.

## Conclusions

Mortality and disability rates within a year after sustaining first ever stroke is high. Severe stroke and presence of disability contributed to the high mortality. Financial constraints were a reason for patients’ limitation for follow up clinics and rehabilitation services. Availability of outreach and home based care services are alternative measures for monitoring and managing patients with disability and ultimate reduction in mortality.

## Abbreviations

CT Scan: Computed Tomography Scan
DALYS: Disability Adjusted Life Years
ILO: International Labour Organization
LMICs: Low and Middle Income Countries
MAMC: MUHAS Academic Medical Center
mRS: Modified Rankin Scale
MUHAS: Muhimbili University of Health and Allied Sciences
NIHSS: National Institutes of Health Stroke Scale
SSA: Sub Saharan Africa
WHO: World Health Organization
